# Diffusion kurtosis imaging in acute ischemic stroke: A systematic review of clinical correlations and prognostic utility

**DOI:** 10.1007/s00234-025-03822-8

**Published:** 2025-10-27

**Authors:** Tancia Pires, Priyanka, John M Solomon, Rajagopal Kadavigere, Saikiran Pendem, Priya P S, Nikith A Shetty

**Affiliations:** 1 Department of Medical Imaging Technology, Manipal College of Health Professions, Manipal Academy of Higher Education, Manipal, Karnataka 576104 India; 2 Department of Physiotheraphy, Manipal College of Health Professions, Manipal Academy of Higher Education, Manipal, Karnataka 576104 India; 3 Center for Comprehensive Stroke Rehabilitation and Research, Manipal Academy of Higher Education, Manipal, Karnataka 576104 India; 4 Department of Radiodiagnosis and Imaging, Kasturba Medical College, Manipal Academy of Higher Education, Manipal, Karnataka 576104 India; 5 Department of Neurology, Kasturba Medical College, Manipal Academy of Higher Education, Manipal, Karnataka 576104 India

**Keywords:** Acute ischemic stroke, Diffusion kurtosis imaging, Stroke outcome, Prognosis prediction

## Abstract

**Purpose:**

Accurate early prediction of long-term disability in acute ischemic stroke remains challenging, necessitating sensitive imaging biomarkers. Diffusion Kurtosis Imaging (DKI) is an emerging modality with high sensitivity, potentially offering numerous diffusion-based prognostic markers for various stroke outcomes, which we aim to establish via this systematic review.

**Methods:**

Five databases were searched to obtain records of studies that were performed in hemispherical, first-ever acute ischemic stroke patients from the inception of the database until January 2025. Studies assessing the ability of DKI to predict various motor, functional, tissue, or neurological outcomes were included. The quality of each study was assessed via the Quality of Prognostic Studies (QUIPS) tool. Two independent reviewers performed two-step screening and data extraction, and the data were synthesized, qualitatively assessed, and reported.

**Results:**

Out of 215 records, 11 studies were included in the review, with 550 total samples diagnosed with acute ischemic stroke who were followed up for a period ranging from 2 to 365 days, with a mean age of 58 ± 6.3 years. Motor outcomes were assessed in 1 study, functional outcomes in 4 studies, tissue-related changes in 2 studies, and neuropsychiatric sequelae in 4 studies. All the studies were of moderate to high quality and thus had a low risk of bias. Among the DKI parameters, Mean Kurtosis was correlated with most outcome assessment tools.

**Conclusion:**

Stroke rehabilitation is a resource-intensive process necessitating optimal patient selection. DKI shows promise for predicting early microstructural changes and global functional outcomes, especially using the Mean Kurtosis (MK) parameter, though evidence for motor-specific recovery remains limited.

**Supplementary Information:**

The online version contains supplementary material available at 10.1007/s00234-025-03822-8.

## Introduction

Ischemic Stroke is one of the major causes of long-term disability; of these, motor impairment is the most common [1]. Timely and personalized rehabilitation in the acute stage can reduce the long-term effects on motor function [2]. Neurological deterioration (ND) is yet another devastating complication that is associated with poor outcomes [3]. The outcomes in stroke survivors vary based on the infarct core lesion volume, region, treatment, or rehabilitation strategies selected.

Currently, the imaging modalities used for diagnosis include Computed Tomography (CT) and Magnetic Resonance Imaging (MRI), where Diffusion-weighted imaging is the leading technique of choice for early diagnosis. However, the technique remains suboptimal for comprehensive lesion characterization, leading to neurological deterioration [4]. Certain hyperintensities exhibiting restricted diffusion, especially in the context of early reperfusion, may demonstrate partial or complete reversibility and/or pseudo-normalisation. Consequently, the extent of DWI abnormalities does not invariably correspond to the true ischemic core, potentially resulting in an overestimation of infarct size and misinterpretation of clinical prognosis, especially in the hyperacute phase. Moreover, a false assumption of a larger core may exclude patients from receiving standard clinical care with mechanical thrombectomy [5]. Despite clinical evidence, some patients may show no detectable lesion on DWI, a phenomenon termed DWI-negative stroke, leading to a false negative diagnosis. Advancement of DWI, the Diffusion Tensor Imaging (DTI) assumes Gaussian diffusion, oversimplifying the complex microenvironment of acute ischemia and often missing subtle or heterogeneous changes, especially in grey matter and regions with crossing fibres, and has inferior accuracy and specificity for early outcome prediction [6]. Hence, there is an unfulfilled clinical need for an early imaging marker of microstructural injury that would complement clinical assessment in predicting acute ischemic stroke outcomes and patient response to rehabilitation therapy. Prognostic ambiguity has stimulated interest in identifying stroke recovery biomarkers.

Diffusion Kurtosis Imaging (DKI) is an emerging imaging technique that is an extension of DTI and is considered more sensitive in quantifying microstructural tissue damage and heterogeneity in tissues, as it accounts for the non-Gaussian diffusion process [7]. The most common DKI parameters are as follows: Mean Kurtosis (MK) the average of the diffusion kurtosis along all diffusion directions; Axial Kurtosis (AK), the kurtosis along the axial direction/parallel to the white matter tracts; and radial kurtosis (RK), the kurtosis along the radial direction of the diffusion ellipsoid, perpendicular to the direction of white matter tracts. Increases and decreases in kurtosis indicate potential pathological changes or microstructural abnormalities, providing more information than diffusion coefficient data do [8]. This finding needs to be explored further to understand the feasibility of DKI in predicting various stroke outcomes. Hence, we aimed to identify specific DKI parameters that can potentially serve as biomarkers for a particular outcome after acute ischemic stroke. This would allow clinicians to make informed decisions regarding the appropriate course of treatment and personalized rehabilitation strategies.

## Methodology

The protocol for this systematic review has been registered in PROSPERO (bearing registration number CRD420251028326) and can be openly accessed. It is reported following the Preferred Reporting Items for Systematic Reviews and Meta-Analyses (PRISMA) guidelines [9] A detailed checklist of the same is available in supplementary file S1.

### Literature search

Five online databases, PubMed, Scopus, Web of Science, CINAHL Ultimate, and Embase, were searched from their inception to January 2025 via a comprehensive search strategy created for PubMed with relevant MeSH terms and appropriate Boolean operators. (available as supplementary material S2). The search strategy was then adapted to the other databases. The reference lists of all eligible studies were also screened to identify any additional relevant sources.

## Eligibility criteria

### Inclusion criteria

Studies were included if they met the PICOTS criteria, where the study population consisted of human subjects diagnosed with first-ever unilateral acute ischemic stroke confirmed by MRI. Patients should have undergone the DKI sequence within 48 h of stroke symptom onset. Studies comparing DKI metrics with conventional diffusion imaging techniques (e.g., DWI, ADC, IVIM, DTI), if available, were also included.

Studies were included if they reported baseline DKI parameters as predictor variables and evaluated stroke outcomes at a later time point via clinical assessment tools such as the NIHSS, modified Rankin Scale (mRS), Fugl-Meyer assessment, Barthel Index, Montreal Cognitive Assessment (MoCA), Mini-Mental State Examination (MMSE), or language evaluations. Studies were also eligible if they demonstrated the predictive value of DKI for functional outcomes, including motor, speech, or cognitive recovery. The time frame considered was from the introduction of Diffusion Kurtosis Imaging in 2005 until January 2025. Hospital-based prospective studies, cohort, case-control, or longitudinal studies that fulfilled all the above criteria were included in this systematic review.

### Exclusion criteria

Studies using only animal models or in vitro imaging; case reports, review articles, and editorials; studies without clear clinical correlation or prognostic/functional assessment; studies where DKI was performed beyond the acute stroke phase; and studies with no available full-text or non-translatable foreign language articles.

## Data selection and extraction

All the retrieved reports were uploaded to Rayyan.ai software for deduplication and screening. Two reviewers (TP and PR) independently followed a two-step study selection screening process on the basis of the pre-determined eligibility criteria. Step one, involved title and abstract screening, followed by step two, full-text screening. The third senior reviewer (RK) resolved any conflicts of interest. Two reviewers (TP and PR) extracted study-relevant data independently, including study characteristics (first author name, date of publication, country), patient demographic data (sample size, age, sex, stroke symptoms, symptom onset time, laterality, etc.), image acquisition parameters (DKI, DWI parameters), clinical correlation results, and/or parameters related to the predictive performance of DKI. Any disagreements were resolved by a senior reviewer (JSM) during data extraction, who approved the final data extraction sheet.

## Assessment of quality (Risk of Bias)

Study quality and risk of bias (RoB) were assessed by two independent reviewers (TP and PR) using the Quality of Prognostic Studies (QUIPS) tool. The QUIPS tool consists of six important domains that can be critically appraised when evaluating validity and bias in studies of prognostic factors. A conclusive judgment within each domain was made and expressed on a three-point scale: high, moderate, or low RoB. Any disagreements were resolved by consensus.

## Data synthesis

The findings from the included studies were synthesized narratively. Owing to the heterogeneity in study designs and the use of diverse assessment tools for evaluating various stroke outcomes, a meta-analysis was not feasible.

## Result

### Literature search results

**Fig. 1:**
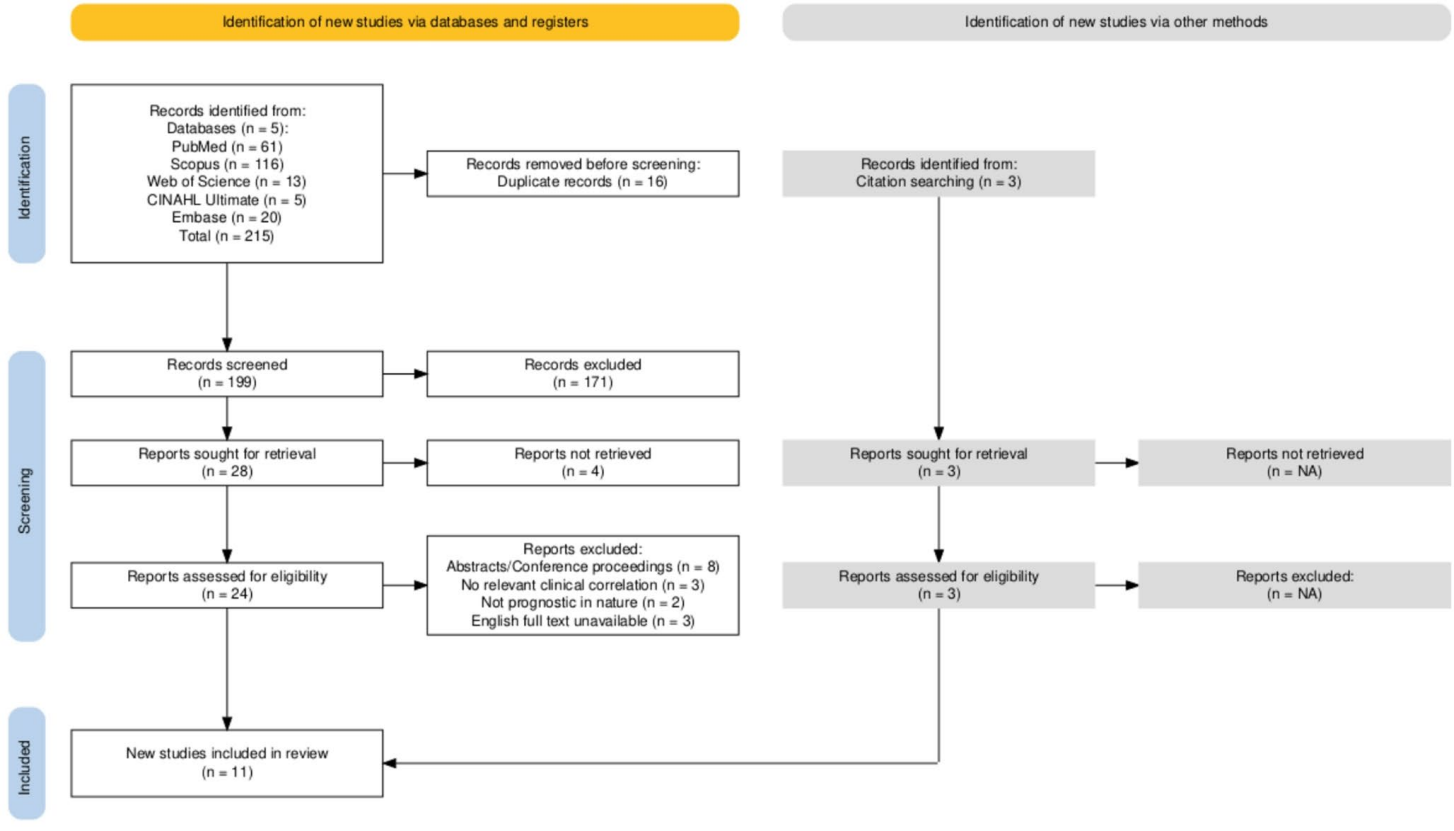
A PRISMA flow diagram depicting the study selection process

A total of 215 records were identified from five databases, 16 of which were duplicates. A total of 171 studies were excluded during title and abstract screening, while 16 were excluded after full-text screening for the reasons mentioned in the PRISMA flow diagram (Fig. 1). Another 3 studies were identified through citation searching or snowballing. A total of 11 studies were ultimately included in the qualitative analysis and data synthesis [10–20]. 

## Quality of the included studies

**Fig. 2:**
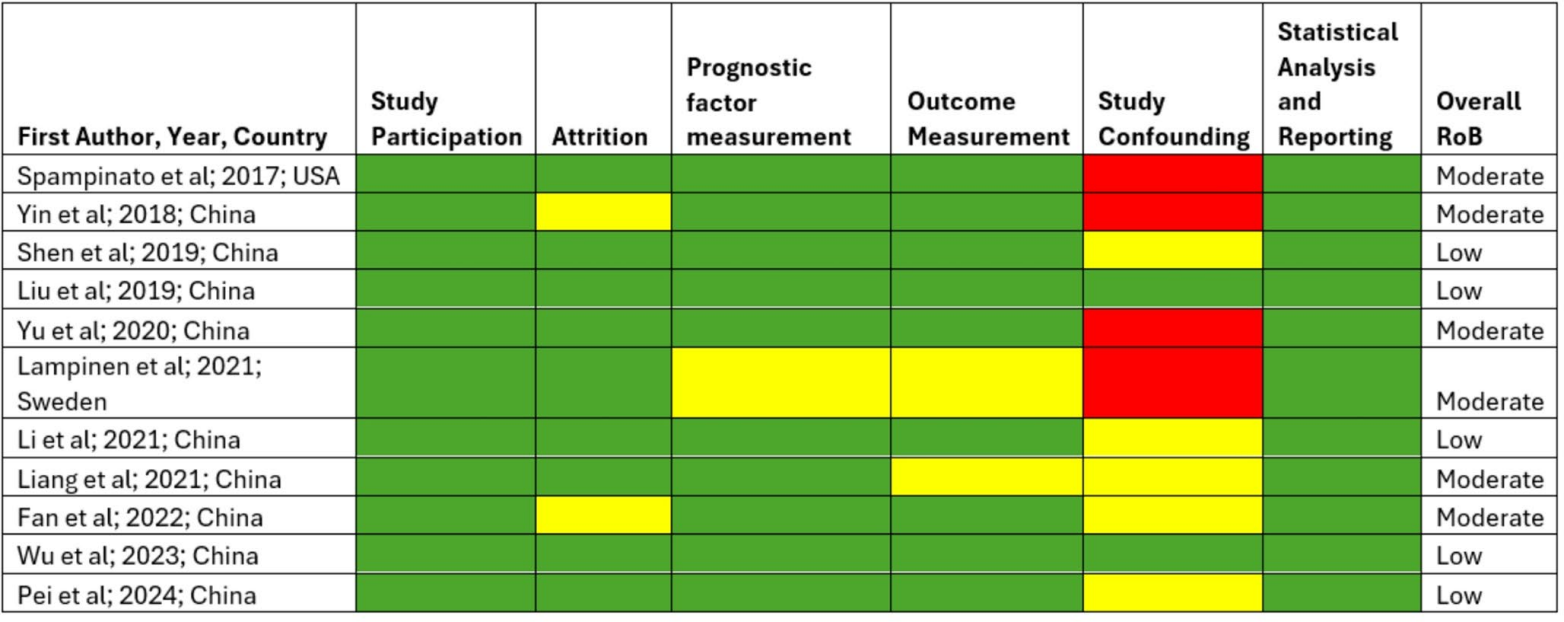
Quality assessment of the included studies via the QUIPS tool

The quality of the studies and risk of bias were assessed via the QUIPS tool. The studies were assessed across six domains, i.e., study participation, attrition, prognostic factor measurement, outcome measurement, study confounding, and statistical analysis and reporting. In each domain, low RoB is denoted in green, moderate or unclear is denoted in yellow, and high RoB is denoted in red. Based on all six domain results, the overall RoB of the study was derived. None of the studies reported high RoB. Six studies had moderate RoB, whereas the remaining five studies had low RoB. A detailed depiction is provided in Fig. 2.

### Study characteristics

The total number of patients from 11 studies was 550, of which more than 50% were males. The mean age was approximately 58 ± 6.3 years. All the studies were prospective in nature, and most were longitudinal, with a follow-up period ranging from 3 weeks to one year after the stroke. Among the 11 studies, one [10] assessed motor outcomes at 3 months poststroke via tools such as the FMA and NIHSS. Four [12, 13, 16, 17] studies assessed functional outcomes via mRS and the Barthel index. Two [14, 18] studies estimated tissue outcomes, whereas four [11, 15, 19, 20] others evaluated the poststroke neuropsychiatric sequelae which included behavioral changes, cognitive impairment, poststroke depression or anxiety, language recovery outcomes, attention and executive functions, which were evaluated via tools such as the MoCA, MMSE, and standardized language assessments. The DKI parameters assessed were MK, AK, RK, and Kurtosis Anisotropy (KA).

### Correlations between clinical parameters and DKI parameters

Motor and functional outcomes assessed via the Fugl-Meyer upper extremity (FM-UE) scale, NIHSS, and mRS at 90 days showed strong negative correlations with lesion-site MK (*r* = − 0.72) and AK (*r* = − 0.75), indicating that lower kurtosis values in the lesion were associated with greater motor impairment [10]. In another study using T2-weighted imaging at 30 days, the MK in the lesion exhibited a strong positive correlation (*r* = 0.730) with outcome, highlighting its potential predictive value [18]. 

Depression severity measured by the HAM-D24 (24-item Hamilton Depression Rating Scale) at 21- and 14 days poststroke was associated with region-specific MK and RK values. Significant negative correlations were detected in several brain regions, including the left and right superior frontal (SF), medial frontal (MF), and temporal (T) lobes, as well as the genu of the corpus callosum (G-CC). MK values ranged from *r* = − 0.346 to − 0.717, whereas RK values ranged from *r* = − 0.340 to − 0.676, reflecting the relationship between microstructural damage and depressive symptoms [15]. 

The cognitive outcomes assessed by the MMSE and MoCA showed region-specific associations with DKI parameters. Notably, the MK, AK, and RK values in areas such as the frontal and parietal lobes, corpus callosum, caudate nucleus, and internal capsules were identified as relevant predictors. In a year-long follow-up, Kurtosis Fractional Anisotropy (KFA) and MK values were linked to long-term cognitive outcomes [20]. 

Infarct progression over time was also studied. Elevated MK values during the acute stage (days 2 and 9) were associated with infarction presence at 100 days, highlighting the potential utility of DKI for early prediction of lesion evolution [14]. The detailed study characteristics and the predictive biomarkers are presented in Table 1.

**Table 1:** Characteristics of the included studies

Sr. No.	First Author, Year, Country	Sample size	Males	Age	Outcome assessed	Outcome assessment tool used	follow-up duration (days)	DKI Predictors
1	Spampinato et al.; 2017; USA [10]	17	11	55.7 ± 12.3	Motor	FM-UE scale, NIHSS, and mRS	90	MK (*r*=−0.72), AK (*r*=−0.75) in lesion
2	Yin et al.; 2018; China [18]	37	28	58 ± 11.8	Tissue (Infarct volume)	T2 weighted imaging	30	MK in lesion (*r* = 0.730)
3	Shen et al.; 2019; China [11]	58	30	64 ± 10.95	Neuropsychiatric sequelae	HAM-D24	21	#MK in frontal, temporal lobes, and genu of CC
4	Liu et al.; 2019; China [12]	80	62	56.3 ± 11.6	Functional	mRS	180	MK in lesion (*r* = 0.345)
5	Yu et al.; 2020; China [13]	48	26	58.27 ± 12.89	Functional	mRS	90	No significant correlations.
6	Lampinen et al.; 2021; Sweden [14]	5	2	47 ± 10	Tissue (infarcted/viable)	DWI	2, 9 and 100	#Elevated MK in acute stage implied infarction at 100 days.
7	Li et al.; 2021; China [16]	48	23	57.1 ± 10.3	Functional	mRS	90	MK (AUC = 0.785) AK (AUC = 0.774)
8	Liang et al.; 2021; China [15]	38	30	64.9 ± 9.72	Neuropsychiatric sequelae	HAM-D24	14	**MK** in L-SF (*r* = − 0.489) R-SF (*r* = − 0.510) L-MF (*r* = − 0.717) R-MF (*r* = − 0.346) L-T (*r* = − 0.66) R-T (*r* = − 0.602) G-CC (*r* = − 0.668) RK of L-SF (*r* = − 0.340) L-MF (*r* = − 0.490) R-MF (*r* = − 0.457) L-T (*r* = − 0.467) R-T (*r* = − 0.676)
9	Fan et al.; 2022; China [19]	70	15	63.1	Neuropsychiatric sequelae	MMSE	NA	**MK** in L-F, R-P, S-CC, B/L CN; ALIC; PLIC; T **AK** in R-F; B/L-CN; B/L-T **RK** in R-F; S-CC; R-CN; B/L-T
10	Wu et al.; 2023; China[20]	60	42	57.70 ± 7.33	Neuropsychiatric sequelae	MMSE, MoCA	365	KFA and MK
11	Pei et al.; 2024; China[17]	89	68	61.8 ± 9.71	Functional	mRS	90	rMK (*r* = 0.545)

*FMA-Fugl-Meyer Assessment; mRS- modified Rankin Scale; MK- mean Kurtosis; AK-Axial Kurtosis; HAM-D- Hamilton Depression Scale; DWI-Diffusion weighted imaging; G-genu’ S-Splenium; CC-Corpus Callosum; R – right; L-Left; SF- Superior frontal gyrus; MF- Middle frontal gyrus; T- Temporal lobe; MMSE = Mini Mental State Examination; B/L-Bilateral; CN-Caudate Nucleus; A/PLIC-Anterior/Posterior limb of internal capsule; MoCA-Montreal Cognitive Assessment; KFA-Kurtosis Fractional Anisotropy; r-relative.*

*# correlation analysis not reported.*

## Discussion

This systematic review successfully describes the potential of DKI parameters in predicting several acute ischemic stroke outcomes, which include motor, tissue, functional, and neuropsychiatric outcomes.

### Motor outcomes

One of the first prediction studies among the 11 studies included in this review was performed by Spampinato et al. in 2017 and was the only study that focused specifically on motor ability. This highlights the limited availability of motor-specific evidence. The authors assessed the value of DKI in assessing and potentially predicting motor outcomes at 3 months poststroke and reported that DKI-derived metrics are more sensitive than DTI in assessing early cortico-spinal tract (CST) microstructural changes. The outcome assessment tools used were the FMA-UE, mRS, and NIHSS at baseline and at 3-month follow-up, where the acute lesion parameters MK and AK were strongly correlated with the FMA scores. A moderate correlation was also found between lesion volume and the FM-UE score at 3 months poststroke. However, the DKI parameters are more predictive of long-term motor impairment than lesion volume alone and could be potential biomarkers; however, owing to the relatively small sample size, the results require validation. Moreover, only one study has reported motor-specific outcomes, which, in fact, is one of the most common ischemic stroke outcomes. This highlights the need for more longitudinal studies in this context [10]. 

### Functional outcomes

Functional outcomes are usually evaluated via a mRS, which is an ordered scale coded from 0 (no symptoms at all) to 5 (severe disability) or 6 (death) [21]. One of the first studies to evaluate the correlation between the diffusion dynamics of ischemic lesions and functional outcomes at 6 months poststroke was performed by Liu et al. in 2019. The largest percentage change between the lesion area and the non-lesion area was observed for the MK, and when the lesion volume was controlled, the MK ratio moderately correlated with the functional outcome [12]. Another study assessed not only the lesion but also the DKI parameters in the CST beyond the lesion via voxel-wise and slice-wise analysis instead of typical ROI analysis in the acute stage and functional outcome correlation at 3 months poststroke. They reported that the relative Axial kurtosis (rAK = affected CST - unaffected CST/unaffected CST) independently correlated with the baseline m-NIHSS score (β = 0.297, *P* = 0.040). However, the long-term correlation between DKI parameters and the mRS score was not significant [13]. The severity of motor impairments is correlated with functional disability and quality of life. However, functional outcome is a broader term for overall outcomes beyond motor outcomes, which could be influenced by a multitude of factors, including rehabilitation, comorbidities, and lifestyle changes [22]. These variables could have perhaps diluted the direct impact of DKI parameters.

Furthermore, Li et al. also assessed CST injury via lesion load analysis which combines DKI-based tract segmentation and orientation mapping, convolutional neural networks, and CST tractograms. It is an innovative technique devoid of subjectivity due to human interaction with increased reproducibility. This study revealed that the MK was significantly correlated with initial motor impairment and functional recovery, with a sensitivity of 0.846 and specificity of 0.682 (*p* = 0.008). Additionally, the AK had high sensitivity (0.923) but relatively low specificity (0.636) in distinguishing between good and poor outcomes (*p* < 0.004). These findings highlight the effectiveness of the tractometry-based image processing and analysis techniques [16]. 

Pei et al. assessed multiple clinical and imaging parameters that could aid as prognostic biomarkers in AIS. The results concerning DKI parameters revealed that the relative Mean Kurtosis (rMK), the ratio between the lesion and contralateral normal value, had the strongest correlation with the mRS score at 90 days postdischarge (*r* = 0.545, *p* < 0.001), whereas the relative Mean Diffusivity (rMD) had a negative correlation. ROC analysis revealed that the rMK had the highest predictive accuracy for AIS outcomes, with an AUC of 0.815 [17]. 

Although motor impairment is a hallmark of post-stroke disability, only one included study directly evaluated motor outcomes, whereas most others relied on global functional scales (e.g., mRS). Our findings, therefore, reflect a broader functional perspective, with limited motor-specific evidence. Future studies incorporating detailed motor assessments are warranted to better delineate the predictive utility of imaging biomarkers for motor recovery.

### Tissue outcomes

**Fig. 3:**
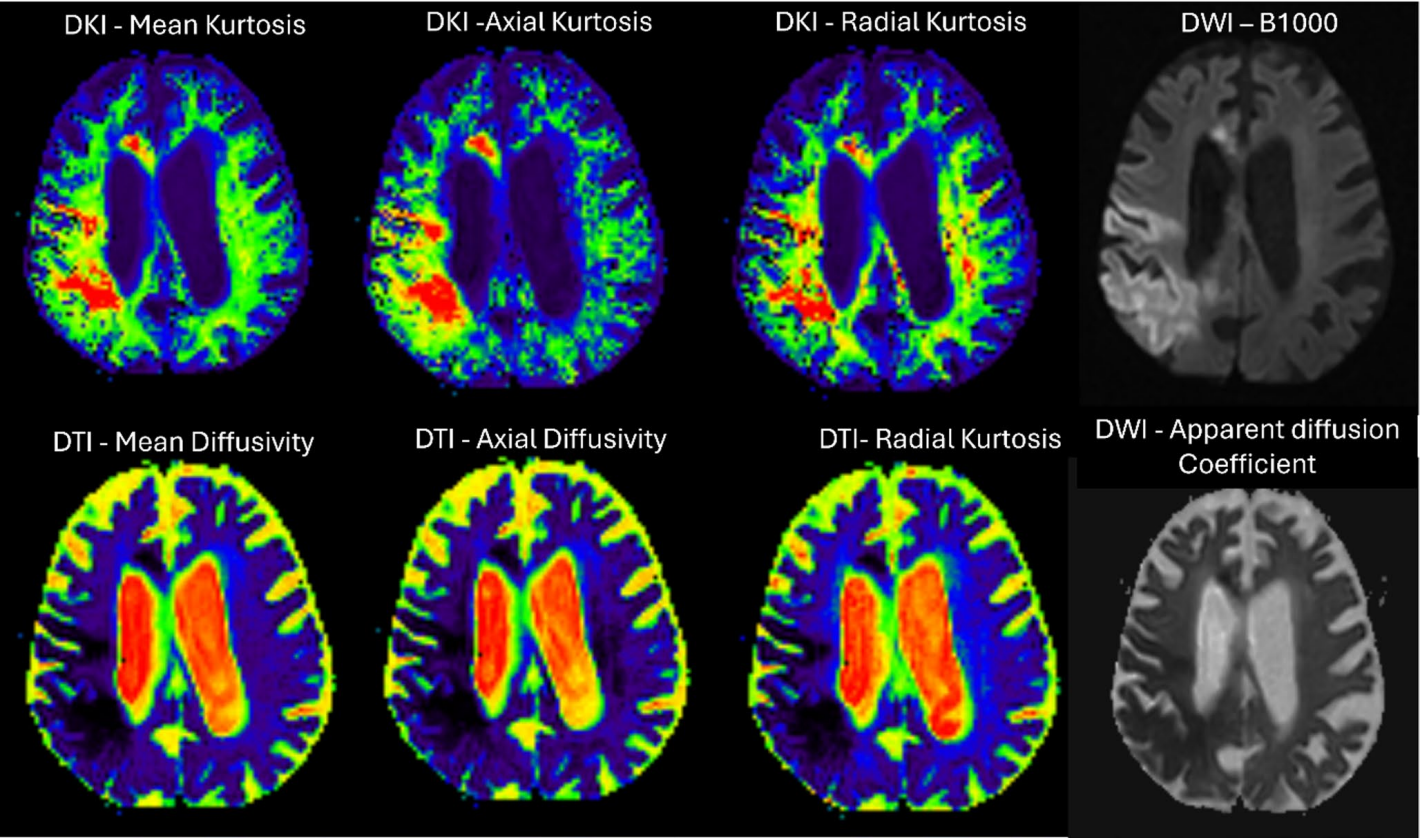
Representative DWI, DTI, and DKI maps of an 86 y/o female with an acute ischemic stroke in the left parietal lobe. Image courtesy of Department of Radiodiagnosis and Imaging, Kasturba Medical College, Manipal. Patient scanned using United Imaging uMR780 3 T scanner

Currently, Acute ischemic Stroke is imaged using DWI and the corresponding ADC maps; however, these are inadequate for lesion characterization. Although DTI maps further enable imaging the microstructural integrity, the assumption regarding the Gaussian distribution of water molecules is oversimplified. Hence, DKI maps are known to be more sensitive for lesion characterisation as illustrated in Fig. 3.

Further, tissue outcome evaluations reveal whether brain tissue recovers or progresses to infarction. It aids in predicting the extent, number, and location of infarcted tissue on follow-up images. A study by Yin et al. (2018) determined the relationship between DWI and DKI in patients with acute stroke at admission and the tissue outcome by assessing the NIHSS score and stroke volume on T2-weighted MR images one month after stroke onset. Their study revealed that, for large lesions, the acute MK lesion volume was significantly more strongly correlated with the outcome infarction volume than the lesion volume based on DWI parameters (i.e., ADC and MD). In the case of small lesions, the number of kurtosis lesions was persistent on follow-up imaging. Thus, DKI may be a better marker for predicting tissue outcome at 1 month, with respect to the lesion volume and number of lesions. Although the NIHSS score was assessed in this study, no statistical analysis was performed to establish any relationship between the entities [18]. Lampinen et al. also assessed tissue outcomes by performing repeated brain scans at 2-, 9- and 100-day intervals; their findings were in line with previous findings, where the baseline elevated MK values in the lesion implied infarction at the follow-up stage. A high ADC indicates infarction of 50 ± 20% of the lesion volume. Therefore, MK is more sensitive and a better predictor of tissue outcome. However, this study was performed in a very small cohort, and further validation is needed [14]. 

### Poststroke neuropsychiatric sequelae prediction

Early identification and management of neuropsychiatric sequelae, including poststroke depression and cognitive impairment, are essential for improving long-term outcomes in stroke survivors. Poststroke depression (PSD) is a prevalent neuropsychiatric complication associated with an increased risk of impaired functional recovery, diminished quality of life, and elevated mortality that affects nearly one-third of stroke survivors at various stages post-event. [23] Shen et al. (2019) used the Hamilton depression scale with 24 items (HAM-D24) at 3 weeks poststroke to assess the depression status of acute ischemic stroke patients and classified them into three groups: Poststroke Depression (PSD), without poststroke depression (N-PSD), and healthy controls (NORM). Although a direct correlation analysis between HAM-D and DKI parameters was not performed, they did find significant differences in the MK values of the bilateral frontal lobes, temporal lobe, and genu of the corpus callosum of the PSD group compared to those of the N-PSD group and the NORM group, which they suggest could be the underlying mechanism of poststroke depression [11]. Another study in 2021 performed with ROI analysis in the limbic-cortical-striatal-pallidal-thalamic (LCSPT) circuit echoed similar results to those of Shen et al., suggesting that the occurrence of PSD may be due to the decrease in complexity caused by the destruction of white matter microstructure in the frontal lobe, temporal lobe, and genu of the corpus callosum. The values of MK and RK in these regions showed significant negative correlations with HAM-D. These findings strengthen the hypothesis that DKI, especially the MK parameter, could be a potential biomarker for predicting the progression of PSD in early settings but requires validation [15]. 

Another impact of acute ischemic stroke could manifest as Vascular Cognitive Impairment (VCI), which is a syndrome comprising language, speech, and calculative disability that can worsen over time and even lead to dementia. Hence, its early diagnosis is important [24]. A study by Fan et al. revealed that DKI-derived parameters may be a viable method to evaluate patients with VCI and to predict potential brain structure imaging biomarkers of VCI progress because DKI values in the bilateral frontal lobe, bilateral parietal lobe, genu and splenium of the corpus callosum, anterior and posterior limb of the bilateral internal capsule, bilateral head of the caudate nucleus, bilateral thalamus, and, bilateral medial temporal lobe, are significantly correlated with baseline MMSE scores. Long-term follow-up and correlation analyses were not performed in this study. However, Wu et al. assessed patients longer than in previous studies, i.e., one-year poststroke for imaging and clinical parametric assessment. This study revealed that lower KFA and MK values in white matter tracts were associated with worse cognitive performance, as measured by baseline MMSE and MoCA scores. The participants were then classified as Poststroke Cognitive Impairment (PSCI) and non-cognitive impairment (NCI). Over a year, patients with PSCI presented faster decreases in KFA and MK values, which were correlated with slower cognitive recovery or further decline. This study also revealed that strategic infarct regions, such as the thalamus and frontal lobes, were strongly correlated with reduced KFA and MK values, highlighting their role in cognitive outcomes. Understanding the specific white matter tracts affected helps tailor cognitive rehabilitation programs to improve functions tied to those regions. Thus, allowing clinicians to predict which patients are at a greater risk of PSCI and may require closer follow-up. Even if cognitive functions improve poststroke, persistent white matter damage detected by DKI suggests a need for ongoing monitoring of these patients. This could help in preventing or managing late-onset cognitive decline [20]. 

## Limitations

This review had a few limitations. First, the review was limited to only five major databases (PubMed, Scopus, Web of Science, Embase, and CINAHL Ultimate). Although this may have excluded some literature, these databases are considered comprehensive and widely used in systematic reviews, minimizing the likelihood of omitting significant studies.

Second, the included studies were restricted to those conducted exclusively in the acute phase of ischemic stroke. While this focus was necessary to maintain consistency with our predefined inclusion criteria, it led to the exclusion of several relevant studies that assessed the utility of DKI in predicting outcomes during the subacute or chronic phases—particularly in relation to speech and language recovery. As a result, the findings may not fully capture the broader applicability of DKI across different stages of stroke recovery. This may be explored further in future studies with broader inclusion criteria. Third, studies that did not conduct formal correlation analyses between DKI parameters and clinical outcomes were excluded. Although some of these studies provided qualitative or exploratory insights into the utility of DKI, they were not eligible under our inclusion criteria, which prioritized quantitative evidence. However, this focus on quantitative evidence enhances the credibility and clinical relevance of our findings. Finally, a meta-analysis was not feasible because of the limited number of studies per outcome and insufficient reporting of numerical data. Therefore, the findings are synthesized narratively, which may limit the statistical power to draw pooled conclusions.

## Conclusion

Conventional clinical predictors may fail to fully account for functional, motor, or neurological recovery variability. Our findings suggest that advanced kurtosis-based parameters, especially Mean Kurtosis, are strongly associated with outcome measures at follow-up, indicating their potential utility in predicting global functional outcomes after acute ischemic stroke. However, given the scarcity of motor-specific evidence, its role in forecasting motor recovery requires further investigation. Patients exhibiting unfavourable diffusion profiles may have limited responses to rehabilitation strategies. However, all these observations need to be further validated in larger cohorts with more extended follow-up periods, and comparative studies with other imaging modalities are deemed necessary, as they underscore the value of imaging-based biomarkers in enhancing patient stratification for future stroke recovery trials.

## Supplementary Information

Below is the link to the electronic supplementary material.

Supplementary Material 1

Supplementary Material 2

## Data Availability

No datasets were generated or analysed during the current study.
