# Supplementary material for: Diffusion kurtosis imaging in acute ischemic stroke: A systematic review of clinical correlations and prognostic utility
Source: Neuroradiology. 2025 Oct 27;67(12):3459–68. doi: 10.1007/s00234-025-03822-8 (PMC12847111; doi:10.1007/s00234-025-03822-8)
Supplement: Supplementary file 2 — Supplementary Material 2 [file 234_2025_3822_MOESM2_ESM.pdf]

Search Strategy For DKI and stroke in Pubmed

Search: **(((Diffusion Kurtosis Imaging) OR (DKI)) OR (Diffusional Kurtosis MRI)) AND ((((Acute ischemic stroke) OR (cerebrovascular accident)) OR (brain stroke)) OR (((("Stroke"[Mesh]) OR "Brain Ischemia"[Mesh]) OR "Ischemic Stroke"[Mesh]) OR (CVA)))** Sort by: **Most Recent**

((("diffusable"[All Fields] OR "diffusant"[All Fields] OR "diffusants"[All Fields] OR "diffuse"[All Fields] OR "diffusely"[All Fields] OR "diffuses"[All Fields] OR "diffusibility"[All Fields] OR "diffusible"[All Fields] OR "diffusion"[MeSH Terms] OR "diffusion"[All Fields] OR "diffused"[All Fields] OR "diffusing"[All Fields] OR "diffusions"[All Fields] OR "diffusive"[All Fields] OR "diffusively"[All Fields] OR "diffusivities"[All Fields] OR "diffusivity"[All Fields]) AND "Kurtosis"[All Fields] AND ("image"[All Fields] OR "image s"[All Fields] OR "imaged"[All Fields] OR "imager"[All Fields] OR "imager s"[All Fields] OR "imagers"[All Fields] OR "images"[All Fields] OR "imaging"[All Fields] OR "imaging s"[All Fields] OR "imagings"[All Fields])) OR "DKI"[All Fields] OR (("diffusional"[All Fields] OR "diffusionally"[All Fields]) AND "Kurtosis"[All Fields] AND ("magnetic resonance imaging"[MeSH Terms] OR ("magnetic"[All Fields] AND "resonance"[All Fields] AND "imaging"[All Fields]) OR "magnetic resonance imaging"[All Fields] OR "mri"[All Fields]))) AND ("Ischemic Stroke"[MeSH Terms] OR ("ischemic"[All Fields] AND "Stroke"[All Fields]) OR "Ischemic Stroke"[All Fields] OR ("acute"[All Fields] AND "ischemic"[All Fields] AND "Stroke"[All Fields]) OR "acute ischemic stroke"[All Fields] OR ("Stroke"[MeSH Terms] OR "Stroke"[All Fields] OR ("cerebrovascular"[All Fields] AND "accident"[All Fields]) OR "cerebrovascular accident"[All Fields]) OR (("brain"[MeSH Terms] OR "brain"[All Fields] OR "brains"[All Fields] OR "brain s"[All Fields]) AND ("Stroke"[MeSH Terms] OR "Stroke"[All Fields] OR "strokes"[All Fields] OR "stroke s"[All Fields])) OR ("Stroke"[MeSH Terms] OR "Brain Ischemia"[MeSH Terms] OR "Ischemic Stroke"[MeSH Terms] OR ("Stroke"[MeSH Terms] OR "Stroke"[All Fields] OR "cva"[All Fields])))
